# Rabies virus monitoring in bat populations in Rondônia state, Brazil

**DOI:** 10.1590/0037-8682-0109-2020

**Published:** 2020-04-22

**Authors:** Marilene Fernandes de Almeida, Adriana Ruckert da Rosa, Luzia Fátima Alves Martorelli, Ana Paula Arruda Geraldes Kataoka, Caroline Cotrim Aires

**Affiliations:** 1 Centro de Controle de Zoonoses, Coordenação de Vigilância em Saúde, Prefeitura da Cidade de São Paulo, SP, Brasil.

**Dear Dr. Jinghui Zhao et al.:**

I would like to thank you for your comments regarding our article entitled “Rabies virus monitoring in bat populations in Rondônia state, Brazil” (https://doi.org/10.1590/0037-8682-0420-2019)^1^. 

This research was conducted to assess the effect of environmental changes on rabies occurrence in bats populations. The samples for this study were frozen and will be analyzed for the presence of lyssavirus and other viruses in the near future at the Rabies Laboratory in Zoonoses Control Center in São Paulo Municipality.

As you noted, there are currently no studies addressing this topic in Brazil, and it is possible that other *Lyssavirus* species are circulating in the country. We therefore aim to test this hypothesis in our study^2^.
